# Exosomal coactivator-associated arginine methyltransferase 1 derived from adipocytes accelerates diabetic wound healing by modulating inflammation and promoting angiogenesis

**DOI:** 10.3389/fbioe.2025.1610806

**Published:** 2025-08-21

**Authors:** Yongxiang Zhang, Yao Pan, Kai Yang, Xiansun Wu, Yaoyao Zhang, Fengbiao Xu, Tietao Di, Wang Liu

**Affiliations:** ^1^ Department of Orthopedics, The 2nd Affiliated Hospital of Guizhou University of Traditional Chinese Medicine, Guizhou University of Traditional Chinese Medicine, Guiyang, China; ^2^ Graduate School, Guizhou University of Traditional Chinese Medicine, Guiyang, China; ^3^ Department of Orthopedics, Ningxiang Hospital of Traditional Chinese Medicine, Ningxiang, China

**Keywords:** adipocyte-derived exosomes, diabetic wound healing, angiogenesis, inflammation, Carm1

## Abstract

**Introduction:**

Delayed wound healing remains a significant clinical challenge under diabetic conditions, characterized by chronic inflammation and impaired angiogenesis. Traditional treatments show limited efficacy, highlighting the urgent need for innovative therapeutic approaches.

**Methods:**

This study investigated the therapeutic potential of exosomes derived from subcutaneous adipocytes (Adipo-EVs) using a diabetic mouse model. Adipo-EVs were locally administered to full-thickness wounds, and healing efficiency was evaluated through wound closure kinetics, histopathology (H&E, Masson’s trichrome), immunohistochemistry (Ki67,α-SMA), and molecular analysis (qPCR, proteomics). The role of the enriched protein Carm1 was validated via siRNA knockdown *in vitro* and *in vivo*.

**Results:**

Adipo-EVs significantly accelerated wound closure, increased cellular proliferation, enhanced collagen deposition, and improved myofibroblast differentiation. Mechanistically, Adipo-EVs suppressed pro-inflammatory cytokines (IL-6, TNF-α) while upregulating IL-10 and promoting angiogenesis (elevated CD31^+^ vessels and in vitro tube formation). Proteomic analysis identified Carm1 as a highly enriched mediator in Adipo-EVs. Knockdown of Carm1 abolished the anti-inflammatory and angiogenic effects of Adipo-EVs, leading to impaired wound repair.

**Discussion:**

Our findings demonstrate that exosomal Carm1 critically modulates inflammation and angiogenesis to enhance diabetic wound healing. This study reveals Carm1 as a pivotal therapeutic component of adipocyte-derived exosomes, offering a novel strategy for managing chronic diabetic wounds.

## Introduction

Chronic skin wounds, particularly diabetic ulcers, represent a significant clinical and economic burden. Diabetic foot ulcers affect approximately 18.6 million people worldwide each year and precede about 80% of diabetes-related lower limb amputations, highlighting the urgency of improved therapies (Armstrong et al., 2023). Biologically, diabetic wounds are challenging to heal due to underlying pathologies such as neuropathy, impaired immune function, and vascular insufficiency. Routine wound healing progresses through coordinated phases of inflammation, new tissue formation, and remodeling. In diabetic patients, however, this process is disrupted–inflammation often persists without resolution, and angiogenesis is insufficient to relieve tissue ischemia (Burgess et al., 2021). The result is a wound environment characterized by prolonged inflammatory signaling and poor neovascularization, which impede proper healing.

Effective wound repair requires both controlled inflammation and robust angiogenesis. In the early stages of healing, a transient inflammatory response helps clear debris and fight infection; this must be tempered over time to avoid tissue damage. Concurrently, angiogenesis (the growth of new blood vessels) is essential to replace damaged capillaries and supply oxygen and nutrients to regenerating tissue. Indeed, angiogenesis drives fibroblast and keratinocyte proliferation, collagen deposition, and re-epithelialization–all vital processes for closing a wound (MacLeod and Mansbridge, 2016; Eming et al., 2014; Sindrilaru et al., 2011). When either of these processes is dysregulated (excessive inflammation or inadequate blood vessel formation), wounds can become chronic and non-healing. Therefore, therapies that modulate inflammation and stimulate angiogenesis hold promise for treating chronic wounds (Beidler et al., 2009; Doupis et al., 2009).

In recent years, extracellular vesicles–especially exosomes–have emerged as essential mediators in regenerative medicine. Exosomes are nano-sized (∼40–150 nm) membrane-bound vesicles of endocytic origin secreted by most cell types. They carry diverse molecular cargo (proteins, mRNAs, microRNAs, lipids) and can shuttle these bioactive signals between cells (Xiao et al., 2021). It is well established that exosomes play key roles in cell-cell communication and can modulate inflammation, angiogenesis, and other healing processes (Xiao et al., 2019; Wang et al., 2019). Multiple studies have demonstrated that exosomes derived from stem cells or progenitor cells can accelerate wound healing. For example, exosomes from endothelial progenitor cells were shown to promote cutaneous wound closure by enhancing angiogenesis via the ERK1/2 signaling pathway (Zhang et al., 2016). Similarly, mesenchymal stem cell (MSC)-derived exosomes recapitulate many of the pro-regenerative functions of the parent MSCs and have shown encouraging results in chronic wound models (Xiao et al., 2019). These findings underscore the potential of exosome-based, cell-free therapies in wound repair.

Despite the growing interest in exosome therapeutics, exosomes derived specifically from adipocytes (adipocyte-derived exosomes, or Adipo-EVs) have been relatively unexplored in the context of skin wound healing. Adipocytes, the chief cells of adipose tissue, are an abundant and easily obtainable cell type (e.g., via liposuction of subcutaneous fat). They are also highly secretory cells, producing an array of signaling molecules–adipokines such as adiponectin and leptin, as well as cytokines, lipids, and various RNAs (Sano et al., 2014; Kranendonk et al., 2014). Many of these factors can be packaged into exosomes. Indeed, adipocyte-derived exosomes have been shown to contain adiponectin (an anti-inflammatory, pro-metabolic cytokine), along with inflammatory mediators like TNF-α and others (Sano et al., 2014; Kranendonk et al., 2014), suggesting they can influence metabolic and immune processes in distant cells. Early studies hint at the functional capabilities of Adipo-EVs. For instance, exosomal transfer of a specific circular RNA from adipocytes was found to trigger inflammation and apoptosis in dermal keratinocytes (Zhang et al., 2019), providing one explanation for impaired healing in obesity. On a more positive note, adipocyte-secreted vesicles have been implicated in maintaining vascular homeostasis and even promoting angiogenesis in specific contexts (e.g., ovarian tissue) (Kranendonk et al., 2014). These observations led us to hypothesize that adipocyte exosomes might exert pro-angiogenic and immunomodulatory effects that could be harnessed to improve wound repair.

Based on this rationale, we designed the present study to investigate whether Adipo-EVs can enhance diabetic wound healing and to elucidate the underlying mechanisms. We first isolated and characterized exosomes from adipocytes and tested their effects on wound healing in a diabetic mouse model and on angiogenesis and inflammation *in vitro*. We then performed proteomic profiling of Adipo-EVs and their parent cells to identify candidate molecules that might mediate the exosomes’ biological activity. Coactivator-associated arginine methyltransferase 1 (Carm1) emerged as a strongly enriched exosomal protein.

Carm1 (PRMT4) is an arginine-methyltransferase that modulates both inflammation and angiogenesis in a context-dependent manner. During acute immune activation, it acts as a co-activator of NF-κB, enhancing pro-inflammatory cytokine transcription, whereas pharmacologic or genetic loss of Carm1 dampens this response (Zhang et al., 2021). In persistent or reparative settings, Carm1 has been reported to favour an IL-10–rich, M2-like macrophage phenotype, thereby facilitating resolution of inflammation (Zhang et al., 2021). In addition, endothelial Carm1 upregulates VEGF through Y-box-binding protein-1 (YB1), promoting angiogenesis and functional blood-flow recovery in ischemia models (Yan et al., 2021). Such dual immunomodulatory and pro-angiogenic capacity makes exosome-delivered Carm1 an attractive strategy for diabetic-wound repair, which simultaneously requires suppression of chronic inflammation and stimulation of neovascularisation.

Carm1 is also known to regulate adipocyte differentiation and metabolic gene expression (Suresh et al., 2021; Yadav et al., 2008), suggesting that adipocytes could be a natural source of Carm1-rich vesicles. We therefore focused on Carm1 as a potential effector of Adipo-EVs. The objectives of this study were to determine if adipocyte-derived exosomes can accelerate the healing of diabetic wounds and to clarify how they do so, with particular attention to the role of exosomal Carm1 in modulating inflammation and angiogenesis during the repair process.

## Materials and methods

### Ethics approval and animals

All animal procedures were approved by the Animal Research Committee of the Second Affiliated Hospital of Guizhou University of Traditional Chinese Medicine. Six-to eight-week-old female C57BL/6 mice (20–25 g) were used in the experiments. Mice were housed under standard laboratory conditions with a 12-h light/dark cycle and *ad libitum* access to food and water. All experiments followed the ethical guidelines for the care and use of laboratory animals.

### Isolation and culture of subcutaneous adipocytes

6-week-old male C57BL/6 mice were euthanized, and subcutaneous adipose tissue from the groin region was dissected and minced into 1–2 mm^3^ pieces. The tissue was digested at 37°C for 30 min in DMEM containing 1% BSA and 25 μg/mL Liberase, with gentle shaking. The digestion was terminated by adding DMEM supplemented with 5% FCS. The suspension was centrifuged at 100 × g for 20 min to separate floating adipocytes, which were transferred to T25 flasks containing serum-free DMEM with 1% BSA. After 24 h, the conditioned medium was collected and stored at −80°C for subsequent exosome isolation (Curtin et al., 2025).

## Isolation and identification of Adipo-Exos

The adipocyte-conditioned medium was centrifuged at 300 × g for 10 min and 2,000 × g for 30 min at 4°C to remove dead cells and debris. The supernatant was then centrifuged at 4,000 × g for 30 min, filtered through a 0.22 µm membrane (Millipore, Billerica, United States), and concentrated using an Amicon Ultra-15 Centrifugal Filter Unit (100 kDa; Millipore) at 4,000 × g. After washing twice with PBS, ExoQuick Exosome Precipitation Solution (System Biosciences, United States) was added, mixed and incubated overnight at 4°C. The mixture was centrifuged at 1,500 × g for 30 min, and the exosome pellet was resuspended in PBS. Exosome purity was confirmed by Western blot using CD63, TSG101, and CD9 as markers (Thery et al., 2006).

## Establishment of a type 1 diabetic mouse full-thickness skin wound model

Type 1 diabetes was induced by intraperitoneal injection of streptozotocin (STZ, 50 mg/kg) for five consecutive days (Wu and Yan, 2015). Mice with blood glucose ≥16.7 mmol/L were confirmed as diabetic. Full-thickness excisional skin wounds (12 mm) were created on the dorsum. Adipo-EVs (200 μg dissolved in 100 μL PBS) or PBS were injected around the wound, and wound healing was assessed on days 3, 5, 7, and 9 post-wounding. Mice were sacrificed at designated endpoints for histopathological analyses.

To explore the role of Carm1 in Adipo-EVs-mediated healing, adipocytes were transfected with control or *Carm1*-targeting siRNA (si-*Carm1*), and Adipo^si−*Cont*^-EVs or Adipo^si−*Carm1*^-EVs were injected into the wounds.

## Evaluation of wound closure

Wounds were photographed on days 3, 5, 7, and 9 post-surgery. Wound areas were quantified using Image-Pro Plus software (Media Cybernetics, Bethesda, United States). Wound-size reduction was calculated as: wound-size reduction (%) = (A_0_ – A_t_)/A_0_ × 100, where A_0_ is the initial wound area, and A_t_ is the wound area on days 3, 5, 7, or 9.

## Evaluation of mechanical tensile strength

On day 9, wound tissue was excised, and tensile stress was measured using a universal testing machine (Instron, United States). Full-thickness skin samples from the wound site were excised, and the tensile stress was measured by applying a stretching force to the wound edges until failure. The maximum tensile stress (in MPa) was calculated based on the force required to break the tissue relative to the cross-sectional area of the wound.

### Histological and immunohistochemical analysis

Skin samples were fixed, embedded in paraffin, and sectioned at 10 µm. H&E staining was performed to evaluate tissue morphology. Masson’s trichrome staining was used to assess collagen density. Sections were subjected to immunohistochemistry (IHC) with primary antibodies against Ki67 (1:500, cat. #A25399, Abclonal), CD31 (1:500, cat. #A19014, Abclonal), IL-10 (1:100, cat. #A2171, Abclonal), IL-6 (1:100, cat. #ab290735, Abcam), or TNF-α (1:1000, cat. #ab307164, Abcam). After washing, the sections were incubated with the corresponding secondary antibodies (1:250, Abcam) using a DAB detection kit. Vessel density and protein expression were quantified in at least three random fields.

### Cell culture

Human microvascular endothelial cells (HMECs; Cell Bank of the Chinese Academy of Sciences, Shanghai, China) were cultured in MCDB131 medium (Gibco) containing 10% fetal bovine serum (FBS), 1 μg/mL hydrocortisone (Sigma), 1% GlutaMAX (Gibco), and 10 ng/mL epidermal growth factor. The murine macrophage cell line RAW264.7 (Cell Bank of the Chinese Academy of Sciences, Shanghai, China) was cultured in high-glucose Dulbecco’s Modified Eagle Medium (DMEM, Gibco) supplemented with 10% FBS and 1% penicillin-streptomycin (Gibco). All cells were maintained at 37°C in a humidified atmosphere containing 5% CO_2_.

### Exosomes uptake assay

Adipocytes were labeled with the green fluorescent dye PKH67 (Sigma) according to the manufacturer’s instructions. The labeled cells were centrifuged at 300 × g for 15 min, and the supernatant was discarded. The cells were washed twice with PBS and seeded into culture flasks for 48 h. Next, Adipo-Exos were isolated from the conditioned medium of these labeled adipocytes and subsequently incubated with HMECs and RAW264.7 cells at 37°C for 3 h. Following incubation, the cells were washed with PBS, fixed with 4% PFA for 15 min, and then washed again with PBS. The nuclei were stained with DAPI (0.5 μg/mL; Invitrogen, Carlsbad, United States). Fluorescence signals were examined using a Leica DMI6000B fluorescence microscope (Solms, Germany).

### LPS-induced inflammatory model

To investigate whether Adipo-EVs could mitigate LPS-induced inflammation, RAW264.7 cells were first seeded into multi-well plates and assigned to one of three treatments for 24 h: (i) uninduced control (phosphate-buffered saline), (ii) LPS alone (100 μg/mL), or (iii) LPS (100 μg/mL) plus Adipo-EVs (100 μg/mL). After incubation, total RNA was extracted for RT-qPCR analysis of inflammatory cytokines (IL-6, TNF-α, IL-10), thereby determining the anti-inflammatory capacity of native Adipo-EVs. Next, to confirm whether this effect was attributable to Carm1, Adipo-EVs were collected from adipocytes transfected with either control or Carm1-targeting siRNA. RAW264.7 cells were again exposed to LPS (100 μg/mL) under these EV treatments (100 μg/mL) for 24 h, followed by RT-qPCR evaluation of the same cytokines.

### Tube formation assay

A Matrigel-based tube formation assay was conducted to determine whether Adipo-EVs enhanced HMEC angiogenesis and to clarify the role of *Carm1* in this process. First, 50 µL of cold Matrigel was dispensed into each well of a 96-well plate and allowed to solidify at 37°C for 30 min. HMECs (1 × 10^4^ per well) were then seeded on the Matrigel surface and treated for 6 h with either PBS or Adipo-EVs (100 μg/mL). Next, to confirm whether Carm1 was responsible for the observed pro-angiogenic effect, HMECs were similarly plated on Matrigel and treated with exosomes derived from adipocytes transfected with either control siRNA (100 μg/mL) or *Carm1*-targeting siRNA (100 μg/mL). After incubation, image analysis software assessed tube formation parameters—such as the number of branching points, total tube length, and loops—under an inverted microscope.

### CCK-8 assay

HMECs and RAW264.7 cells were seeded into 96-well plates (5 × 10^3^ per well) to assess cell proliferation. After the cells adhered, they were exposed for 24 h to treatment. Subsequently, 10 µL of CCK-8 reagent was added to each well, and the plates were incubated at 37°C for 2–3 h. The absorbance at 450 nm was recorded using a Thermo Fisher microplate reader, and the resulting values were used to assess cellular proliferation.

#### Migration assay

To assess the effect of Adipo-EVs on cell migration, Transwell assays were performed using 8-µm pore size inserts (Corning, United States). RAW264.7 cells or HMECs were seeded in the upper chambers of the Transwell inserts at a density of 1 × 10^4^ cells per well in serum-free medium, and the lower chambers were filled with medium containing 10% FBS as a chemoattractant. After 24 h of incubation, the cells that had migrated through the membrane were fixed with 4% paraformaldehyde (Sigma-Aldrich, United States) for 15 min and stained with crystal violet (Sigma-Aldrich, United States) for 30 min. Migrated cells were counted in five random fields per membrane using a light microscope (Olympus, Japan). Results were expressed as the average number of cells per field.

### Quantitative real-time PCR (qRT-PCR) analysis

Total RNA was extracted using TRIzol Reagent (Invitrogen, Carlsbad, United States) and 1 μg total RNA from each sample was reverse transcribed into cDNA with a Revert Aid First Strand cDNA Synthesis kit (Fermentas, Burlington, Canada). qRT-PCR was performed using FastStart Universal SYBR Premix ExTaq (Takara Biotechnology, Japan). Reactions were processed and analyzed on an ABI PRISM^®^ 7900HT System (Applied Biosystems, United States). Relative gene expression was calculated using the 2^−ΔΔCT^ method, and GAPDH was used as a housekeeping gene for normalization. Primer sequences used for qRT-PCR were as follows: *Il6*: forward, 5′-CTGCAAGA GACTTCCATCCAG-3′, and reverse, 5′-AGT​GGT​ATA​GAC​AGG​TCT​GTT​GG-3'; *Il10*: forward, 5′-CTT​ACT​GAC​TGG​CAT​GAG​GAT​CA-3′, and reverse, 5′-GCAGCTCTAGGAGCATGTGG-3'; *Tnfα*: forward, 5′-CAG​GCG​GTG​CCT​ATG​TCT​C-3′, and reverse, 5′-CGA​TCA​CCC​CGA​AGT​TCA​GTA​G-3'; *Carm1*: forward, 5′- AGAGGCTGTGGAATGACAGC-3′, and reverse, 5′- ACA​CCC​ATT​GAG​GCA​AAC​TC-3'; *Gapdh*: forward, 5′-ATC​CCA​TCA​CCA​TCT​TCC-3′, and reverse, 5′-GAG​TCC​TTC​CAC​GAT​ACCA-3’.

### Proteomic sample preparation and TMT labeling

Adipocytes (three biological replicates) were cultured under standard conditions, washed, and then incubated in an exosome-free medium for 48 h. The cells and their secreted extracellular vesicles (EVs) were collected, followed by protein extraction in lysis buffer and subsequent protein precipitation with 15% trichloroacetic acid at −20°C. After resuspending the resulting protein pellets in 8 M urea containing 100 mM tetraethylammonium bromide (pH 8.0), samples were reduced, alkylated, and digested overnight with trypsin. Approximately 100 µg of digested peptide from each sample was then labeled with a 6-plex TMT kit (Thermo Fisher Scientific) according to the manufacturer’s instructions. The labeled peptides were desalted, dried, and fractionated by high-pH reverse-phase high-performance liquid chromatography (HPLC). Jingjie PTM BioLab (Hangzhou, China) performed all proteomic sample processing and labeling steps.

### LC-MS/MS and bioinformatic analysis

Fractionated peptides were separated on an EASY-nLC 1000 system (Thermo Fisher Scientific), analyzed on a Q Exactive™ mass spectrometer (Thermo), and operated in data-dependent acquisition mode. Raw data were processed with MaxQuant (v1.5.2.8), searching against an appropriate species-specific database (SwissProt *Mus musculus*) and maintaining a 1% false discovery rate for proteins, peptides, and PSMs. TMT 6-plex was selected for quantification, with a fold change ≥1.5 set as the threshold for identifying differentially expressed proteins. Gene Ontology (GO) and Kyoto Encyclopedia of Genes and Genomes (KEGG) pathway enrichment analyses (Fisher’s exact test, p < 0.05) were subsequently conducted to classify these proteins into biological processes, molecular functions, and cellular components.

### Statistical analysis

All data are presented as mean ± standard deviation (SD). Statistical analyses were performed using GraphPad Prism 8.0 software. Comparisons between the two groups were analyzed using an unpaired, two-tailed Student’s t-test. In contrast, multiple-group comparisons were conducted by one-way analysis of variance (ANOVA) followed by the Bonferroni *post hoc* test. A two-way ANOVA was also used to compare healing rates among different groups at various time points. A value of P < 0.05 was considered statistically significant.

## Results

### Adipo-EV accelerates cutaneous wound healing in diabetic mice

**FIGURE 1:**
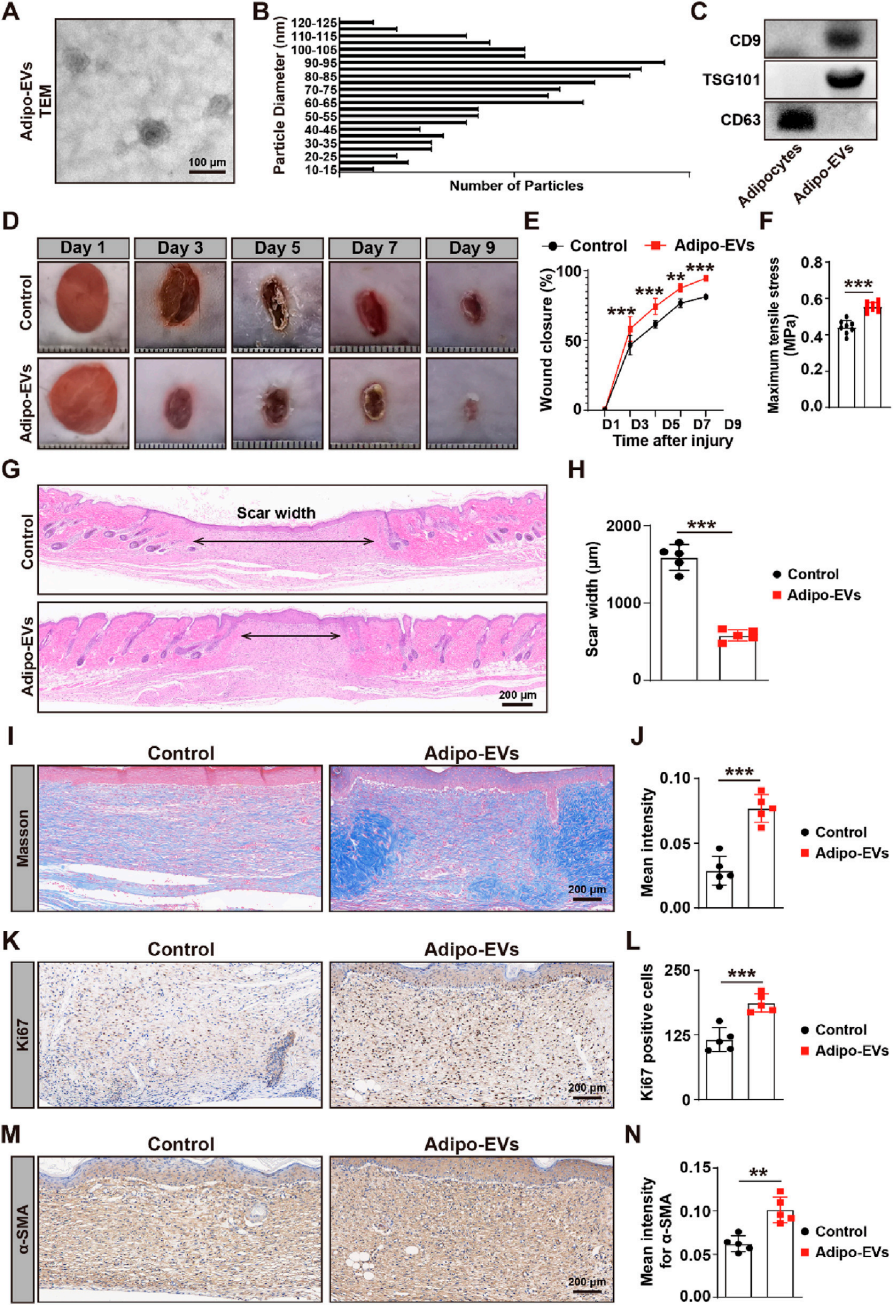
Adipo-EVs accelerate cutaneous wound healing in diabetic mice **(A)** Transmission electron microscopy (TEM) images of purified Adipo-EVs, revealing their characteristic cup- or sphere-shaped morphology. **(B)** Particle size distribution analysis shows that most Adipo-EVs range between 30 nm and 100 nm in diameter. **(C)** Western blot (WB) analysis confirmed the presence of exosomal markers (CD63, CD9 and TSG101). **(D,E)** Representative photographs of full-thickness cutaneous wounds treated with Adipo-EVs or PBS (control), along with quantitative measurements of wound areas on days 3, 5, 7, and 9 post-wounding. N = 5 per group. **(F)** Maximum tensile stress measurements showed significantly higher tissue strength in Adipo-EVs-treated wounds compared to control wounds. **(G,H)** Hematoxylin and eosin **(H,E)** staining on day 9 post-wounding showed narrower scar widths in Adipo–EVs–treated wounds. Quantification of scar width is provided in **(H)**. N = 5 per group. **(I,J)** Masson’s trichrome staining and quantification revealed a greater abundance of well-organized collagen fibers (blue staining) in Adipo–EVs–treated wounds relative to controls. N = 5 per group. **(K, L)** Ki67 immunostaining demonstrates more proliferating cells (brown nuclei) in Adipo–EVs–treated wounds. N = 5 per group. **(M, N)** Immunohistochemical detection of α-smooth muscle actin (α-SMA) indicating increased α-SMA expression in Adipo–EVs–treated wounds, consistent with enhanced myofibroblast differentiation and wound contraction. N = 5 per group. Data are plotted as mean ± SD. ^*^ *P* < 0.05, ^**^ *P* < 0.01, ^***^ *P* < 0.001.

To characterize Adipo-EVs, transmission electron microscopy (TEM) and Western blot (WB) analysis were performed. TEM analysis revealed that Adipo-EVs exhibited a cup- or sphere-shaped morphology (Figure 1A). Particle diameter analysis indicated that the diameters of these particles predominantly ranged from 30 nm to 100 nm (Figure 1B). Concurrently, WB confirmed the presence of exosomal surface markers, including CD63, CD9 and TSG101 (Figure 1C), indicating that the purified nanoparticles were exosomes.

To evaluate the effect of Adipo-EVs on wound healing, full-thickness cutaneous wounds were created on the backs of mice, followed by subcutaneous injection of Adipo-EVs or an equal volume of exosome diluent (PBS). Digital photographs were taken to document wound closure. As shown in Figures 1D,E, wound closure in Adipo-EVs-treated mice was accelerated, with smaller wound areas on days 3, 5, 7, and 9 post-wounding compared to the PBS-treated control group. Mechanical tensile strength measurements revealed that wounds treated with Adipo-EVs exhibited significantly higher tensile strength compared to control wounds, highlighting the beneficial effects of Adipo-EVs on tissue integrity during early healing (Figure 1F). H&E staining showed a narrower scar width in Adipo-EVs-treated wounds compared to those treated with PBS on day 9 post-wounding (Figures 1G,H). Masson’s trichrome staining showed increased amounts of wavy collagen fibers in the Adipo-EVs-treated wounds relative to those treated with PBS (Figures 1I,J). Ki67 immunostaining revealed more Ki67-positive proliferating skin cells in the wounds treated with Adipo-EVs compared to the control group (Figures 1K,L). Immunohistochemistry showed that Adipo-EVs significantly increased α-smooth muscle actin (α-SMA) expression in the wounds by day 9 (Figures 1M,N), indicating that Adipo-EVs treatment accelerates wound healing.

In addition to assessing wound healing, we monitored blood glucose levels at three different time points (D0, D4, and D9) in both the Adipo-EV-treated and control groups. As shown in Supplementary Figure S1, no significant differences in blood glucose levels were observed between the two groups at any time point, confirming that the observed effects on wound healing were not influenced by fluctuations in glucose levels.

### Adipo-EVs inhibit inflammation and promote angiogenesis in the wound sites of diabetic mice

**FIGURE 2:**
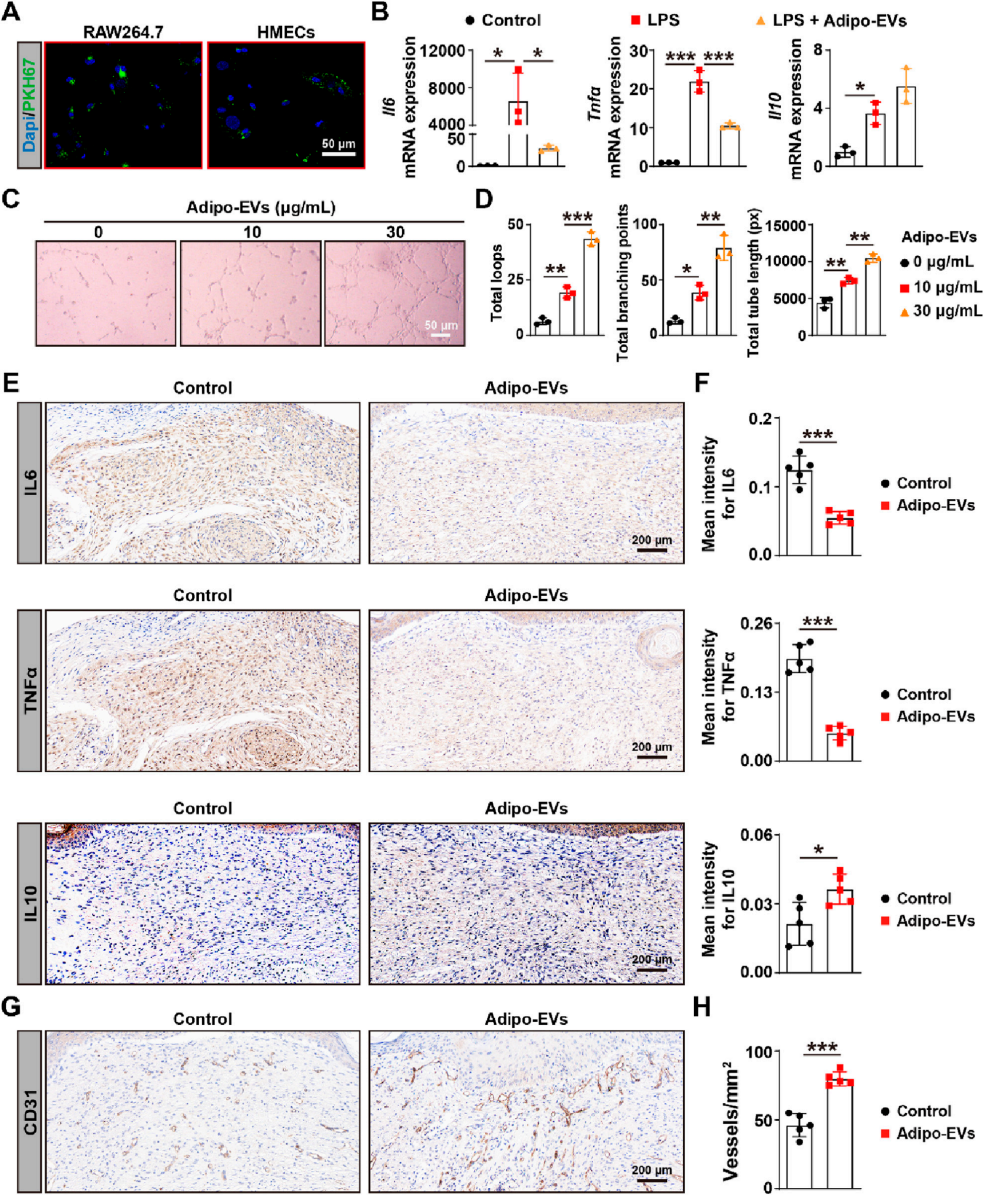
Adipo-EVs inhibit inflammation and promote angiogenesis in mouse wound sites **(A)** PKH67-labeled Adipo-EVs (green) incubated with RAW264.7 macrophages and HMECs. Fluorescence microscopy images demonstrate successful uptake of Adipo-EVs, with signals localized to the perinuclear regions. Nuclei are stained with DAPI (blue). **(B)** RT-qPCR analysis of *Il6*, *Tnfα*, and *Il10* in RAW264.7 cells stimulated with LPS, with or without Adipo-EVs. Adipo-EVs significantly decreased pro-inflammatory cytokines (IL6, TNFα) while increasing IL10, indicating their anti-inflammatory effect. N = 3 per group. **(C,D)** Tube formation assay in HMECs. Representative images **(C)** and quantitative analysis **(D)** (total loops, branching points, and total tube length) show that exposure to Adipo-EVs enhances angiogenic capability. N = 3 per group. **(E,F)** Immunohistochemical (IHC) staining of wound tissues for IL6, TNFα, and IL10. Adipo–EVs–treated wounds exhibit lower IL6 and TNFα levels but higher IL10, suggesting reduced local inflammation. N = 5 per group. **(G,H)** IHC staining for CD31, an endothelial marker, indicates that Adipo-EVs significantly increase the number of CD31-positive vessels in wound areas, thereby promoting angiogenesis. N = 5 per group. Data are plotted as mean ± SD. ^*^ *P* < 0.05, ^**^ *P* < 0.01, ^***^ *P* < 0.001.

Next, we evaluated whether Adipo-EVs could be internalized by the macrophage cell line RAW264.7 and human micro-endothelial cell line (HMECs), which is essential for subsequent experiments. Adipo-EVs were labeled with the green fluorescent dye (PKH67) and incubated with RAW264.7 and HMECs. After 3 h, the cells were washed to remove unbound EVs, fixed, and stained with DAPI. Fluorescence microscopy analysis revealed that the PKH67-labeled exosomes were transferred to the perinuclear region of RAW264.7 and HMECs (Figure 2A). To investigate the impact of Adipo-EVs on LPS-induced inflammatory cytokines gene expression, RT-qPCR was performed to measure IL6, TNFα, and IL10 levels. As shown in Figure 2B, LPS significantly induced mRNA expressions of IL6, TNFα, and IL10 compared to the control group. Adipo-EVs significantly reduced the expression of pro-inflammatory cytokines IL6 and TNFα, while increasing IL10 expression in LPS-induced RAW264.7 cells. In addition, tube formation assay was performed to assess the effect of Adipo-EVs on angiogenesis (Figure 2C). The total number of loops, branching points, and total tube length were measured to quantify the angiogenic capacity of HMECs. As shown in Figure 2D, these indicators were significantly increased upon exposure to Adipo-EVs, suggesting that Adipo-EVs promote endothelial cells angiogenesis.

As inflammation is a critical early response in wounding healing, we further investigated the effects of Adipo-EVs on inflammation. The expression levels of IL6, IL10, and TNFα were analyzed with IHC. The results showed that Adipo-EVs treatment alleviated the inflammatory response by downregulating IL6, TNFα, while upregulating IL10 expression. Finally, to evaluate angiogenesis, dermal microvessels were immunostained for the endothelial marker CD31. As shown in Figure 2G, wounds treated with Adipo-EVs exhibited a higher density of blood vessels compared to the control group. Quantitative analysis of CD31-positive vessels confirmed that Adipo-EVs significantly enhanced angiogenesis in the wound areas (Figure 2H).

### Cell proliferation and migration effects of Adipo-EVs

To assess the potential effects of Adipo-EVs on cell proliferation and migration, CCK-8 and Transwell assays were performed using RAW264.7 and HMECs at concentrations of 10 μg/mL and 30 μg/mL. However, no significant effects on cell proliferation or migration were observed at the tested concentrations of Adipo-EVs (Supplementary Figure S2). As a result, further experiments with *Carm1*-knockdown Adipo-EVs were not conducted.

### Proteomic analysis of Adipocytes and Adipo-EVs

**FIGURE 3:**
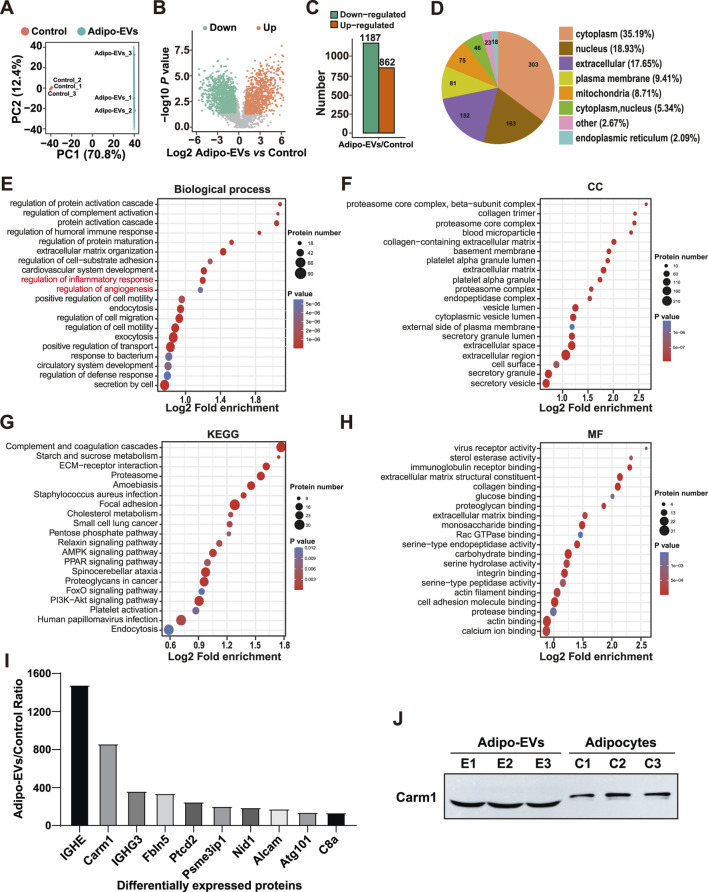
Proteomic analysis of adipocytes and Adipo-EVs **(A)** Principal component analysis (PCA) of proteomic data, showing distinct protein expression patterns between adipocytes and Adipo-EVs. PC1 and PC2 account for 70.8% and 12.4% of the total variance, respectively. **(B,C)** Volcano plot **(B)** and histogram **(C)** depicting differentially expressed proteins in Adipo-EVs, with 1187 downregulated and 862 upregulated (fold change ≥1.5, *p* < 0.05). **(D)** Subcellular localization of differentially expressed proteins, indicating most proteins are found in the cytoplasm (35.19%), nucleus (18.93%), or extracellular components (17.65%). **(E)** Gene Ontology (GO) enrichment analysis of upregulated proteins, highlighting processes such as inflammatory response regulation and angiogenesis. **(F)** Cellular component enrichment analysis shows significant enrichment in the proteasome core complex and collagen-containing extracellular matrix. **(G)** KEGG pathway enrichment analysis reveals involvement in complement and coagulation cascades, ECM–receptor interactions, and *Staphylococcus aureus* infection. **(H)** Molecular function enrichment analysis suggests that many proteins participate in RNA and protein binding and translational regulation. **(I)** The top 10 most abundant proteins in Adipo-EVs, with IGHE and Carm1 displaying the highest fold enrichment, were selected for further study. **(J)** Western blotting to determine the Carm1 protein level in Adipocytes and Adipo-EVs.

To investigate the functional molecules mediating the effects of Adipo-EVs, proteomic analysis was conducted to detect the protein expression profiles in Adipo-Exos and their parent cell adipocytes. A total of 5,475 protein groups were identified, with 3,656 proteins quantified. Differentially expressed proteins were identified with a cutoff of absolute fold change ≥1.5 and *P* value <0.05. Principal component analysis (PCA) revealed a clear distinction between Adipo-EVs and control samples, with PC1 accounting for 70.8% of the variance and PC2 accounting for 12.4% (Figure 3A), indicating significant differences in protein expression. Figures 3B,C show that Adipo-EVs were enriched in 1187 downregulated proteins and 862 upregulated proteins. The cellular localization of the differentially expressed proteins in Figure 3D, with most located in the cytoplasm (35.19%), followed by the nucleus (18.93%) and extracellular components (17.65%), indicating broad effects of Adipo-EVs on multiple cellular compartments. The upregulated proteins in Adipo-EVs were further analyzed for their involvement in biological processes. As shown in Figure 3E, these proteins regulate key processes such as inflammatory response and angiogenesis. Cellular component enrichment analysis (Figure 3F) revealed significant enrichment in structures such as the proteasome core complex and collagen-containing extracellular matrix. KEGG pathway analysis (Figure 3G) highlighted the involvement of upregulated proteins in pathways like complement and coagulation cascades, ECM-receptor interactions, and *Staphylococcus aureus* infection. The proteins were primarily involved in binding activities, including RNA and protein-containing complexes and protein translation (Figure 3H). These results suggest that the upregulated proteins in Adipo-EVs play significant roles in regulating inflammatory responses, angiogenesis, and cellular processes, promoting tissue repair. Figure 3I lists the top 10 most abundant proteins in the Adipo-EVs group, with IGHE and Carm1 showing the highest fold enrichment, both selected for further analysis.

### Carm1 mediates the pro-angiogenic and anti-inflammatory effects of Adipo-EVs *in vitro*

**FIGURE 4:**
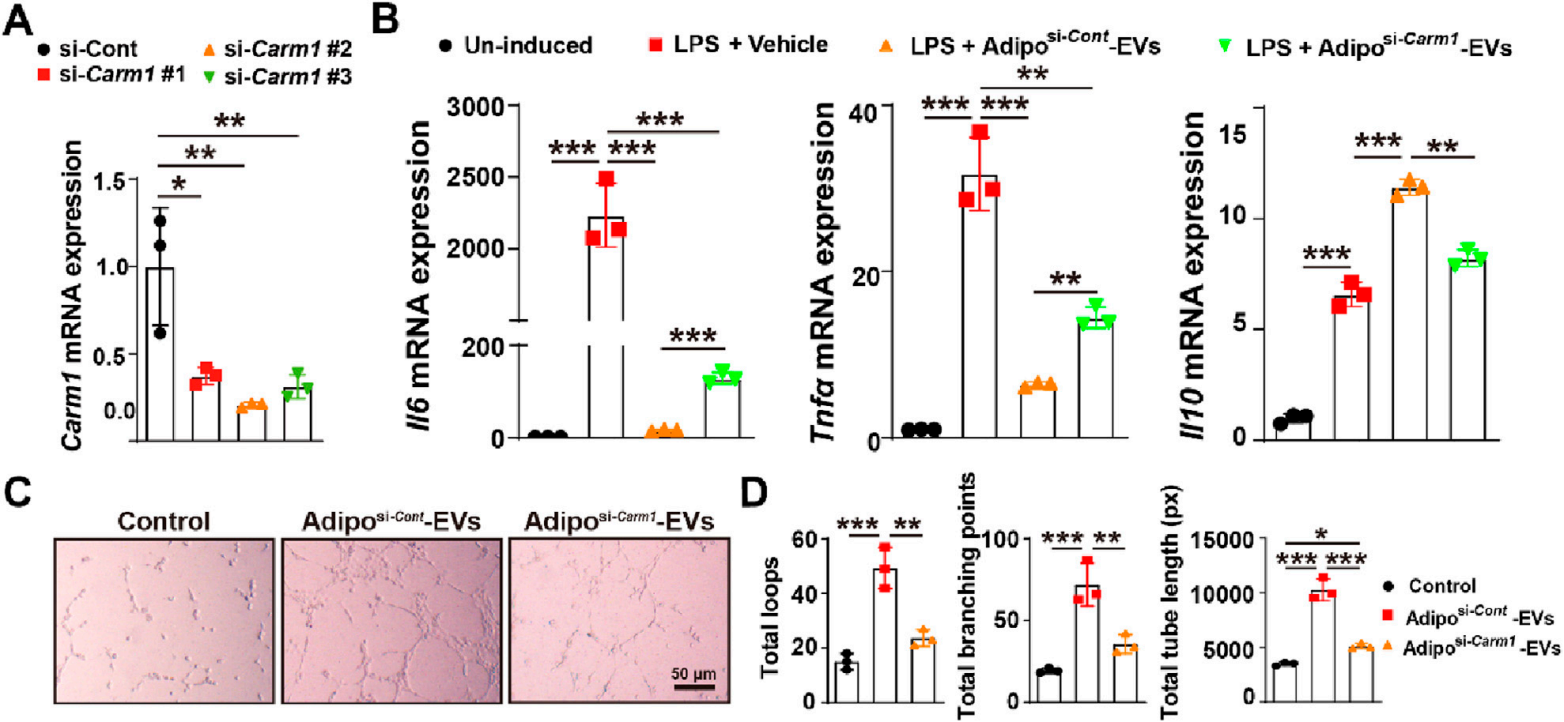
Carm1 mediates the pro-angiogenic and anti-inflammatory effects of Adipo-EVs *in vitro* **(A)** Knockdown of *Carm1* in adipocytes using siRNA (si-*Carm1* #1, #2, #3), as confirmed by qPCR. si-*Carm1*–treated cells exhibit significantly reduced *Carm1* levels compared with control siRNA–treated cells. N = 3 per group. **(B)** qPCR analysis of inflammatory cytokines in cells treated with LPS + vehicle or LPS + Adipo-EVs (si-*Carm1* or control). The results show higher *Il6* and *Tnfα* but lower *Il10* in the si-*Carm1* group, indicating that Carm1 knockdown diminishes the anti-inflammatory activity of Adipo-EVs. N = 3 per group. **(C)** Representative images of HMEC tube formation assays demonstrate fewer capillary-like structures in the si-Carm1 Adipo-EVs group than in the control group. **(D)** Quantitative analysis of total loops, branching points, and tube length confirmed significantly reduced tube formation in the si-*Carm1* group compared with the control group. N = 3 per group. Data are plotted as mean ± SD. ^*^ *P* < 0.05, ^**^ *P* < 0.01, ^***^ *P* < 0.001.

To explore Carm1′role in the pro-angiogenic and anti-inflammatory effects of Adipo-EVs, we knocked down Carm1 in adipocytes using siRNA (si-*Carm1* #1, #2, #3) and confirmed the knockdown efficiency by qPCR. As shown in Figure 4A, si-*Carm1* #2 exhibited the highest knockdown efficiency, and was selected for further experiments. We evaluated the inflammatory response of LPS- treated RAW264.7 cells with Adipo-EVs derived from *Carm1* knockdown or control adipocytes. qPCR analysis revealed that Adipo-EVs from si-*Carm1* adipocytes (Adipo^si−*Carm1*^-EVs) significantly increased the expression of IL6 and TNFα compared to Adipo-EVs from control adipocytes (Adipo^si−*Cont*^-EVs). Conversely, IL10 expression was notably reduced in the si-*Carm1* group (Figure 4B). These results suggest that Carm1 depletion in adipocytes impairs the anti-inflammatory effects of Adipo-EVs. The ability of Adipo-EVs to promote angiogenesis was tested using a tube formation assay with HMECs. Endothelial cells exposed to Adipo^si−*Carm1*^-EVs formed significantly fewer capillary-like structures compared to those treated with Adipo^si−*Cont*^-EVs (Figure 4C). Quantitative analysis revealed a marked reduction in the total number of loops, branching points, and total tube length in the Adipo^si−*Carm1*^-EVs group compared to controls (Figure 4D). These findings underscore the importance of Carm1 in maintaining the pro-angiogenic and anti-inflammatory effects of Adipo-EVs.

### Exosomal Carm1 accelerates cutaneous wound healing in diabetic mice

**FIGURE 5:**
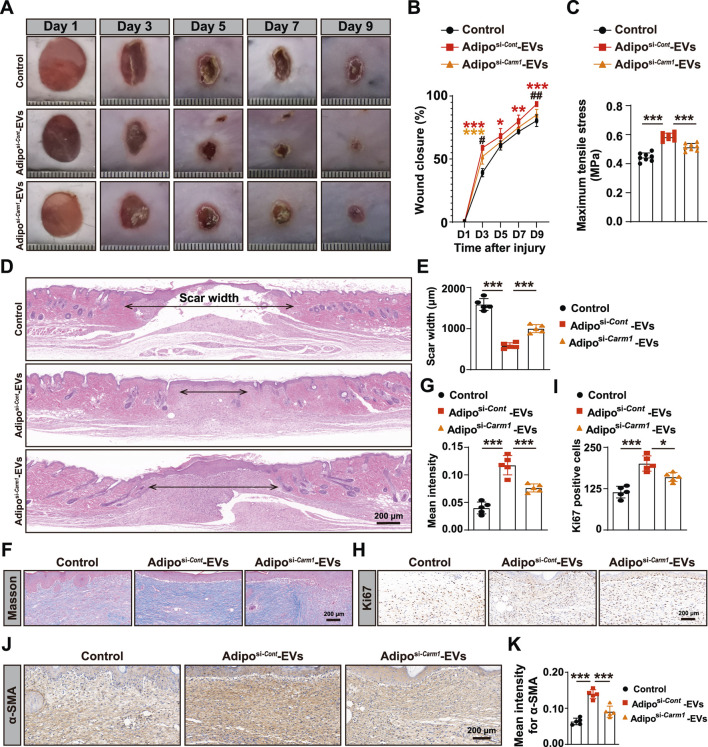
Exosomal Carm1 accelerates cutaneous wound healing in diabetic mice **(A)** Full-thickness cutaneous wounds were created on the backs of mice, followed by subcutaneous injection of Adipo-EVs (si-*Cont* or si-*Carm1*). Wound closure was significantly delayed in the si-Carm1 group, as indicated by measurements on days 3, 5, 7, and 9 post-injury. **(B)** Representative images of wound closure at days 3, 5, 7, and 9. N = 5 per group. **(C)** Maximum tensile stress was significantly higher in wounds treated with Adipo-EVs from control adipocytes compared to those treated with si-*Carm1* Adipo-EVs. **(D)** H&E staining on day 9 post-wounding revealed narrower scar widths in wounds treated with Adipo-EVs from control adipocytes, compared with si-*Carm1* Adipo-EVs. **(E)** Quantification of scar width from H&E-stained sections. N = 5 per group. **(F)** Masson’s trichrome staining shows abundant and more organized collagen fibers in wounds treated with control Adipo-EVs than those treated with si-*Carm1* Adipo-EVs. **(G)** Quantification of collagen density from trichrome-stained sections. N = 5 per group. **(H)** Ki67 immunostaining was used to assess skin cell proliferation at the wound site, with more Ki67-positive cells in control Adipo–EVs–treated wounds than in si-*Carm1*–treated wounds. **(I)** Quantification of Ki67-positive proliferating cells. N = 5 per group. **(J)** α-Smooth muscle actin (α-SMA) staining to evaluate myofibroblast differentiation. Higher α-SMA expression was observed in the control Adipo–EVs–treated wounds. **(K)** Quantification of α-SMA–positive areas. N = 5 per group. Data are plotted as mean ± SD. ^*^ *P* < 0.05, ^**^ *P* < 0.01, ^***^ *P* < 0.001.

To investigate the role of exosomal Carm1 in cutaneous wound healing, full-thickness wounds were induced on the backs of mice, followed by subcutaneous injection of Adipo-EVs from either control si-Cont or si-*Carm1* adipocytes. Figures 5A,B show that wound healing was significantly delayed in mice treated with Adipo^si−*Carm1*^-EVs compared to those treated with Adipo^si−*Cont*^-EVs. Adipo^si−*Cont*^-EVs accelerated wound closure at days 3, 5, 7, and 9 post-injury, while Adipo^si−*Carm1*^-EVs showed a marked reduction in healing efficiency. At all time points, wounds in the Adipo^si−*Carm1*^-EVs group demonstrated significantly larger wound areas compared to the Adipo^si−*Cont*^-EVs group, indicating that Carm1 is crucial for effective wound healing. Mechanical tensile strength measurements (Figure 5C) further confirmed that wounds treated with Adipo^si−*Cont*^-EVs had significantly higher tensile stress compared to those treated with Adipo^si−*Carm1*^-EVs, highlighting Carm1’s role in enhancing tissue strength during early wound healing. Histological analysis revealed that wounds treated with Adipo^si−*Cont*^-EVs exhibited narrower scars compared to those treated with Adipo^si−*Carm1*^-EVs (Figures 5D,E). Masson’s trichrome staining showed more abundant and organized collagen fibers in the wounds treated with Adipo^si−*Cont*^-EVs (Figure 5F), with quantification revealing significantly higher collagen intensity in these wounds (Figure 5G). These results suggest that Carm1 promotes collagen production and organization during wound healing. Ki67 immunostaining (Figures 5H,I) showed significantly more proliferating cells in the wounds treated with Adipo^si−*Cont*^-EVs, suggesting that Carm1 enhances cell proliferation during the healing process. Additionally, α-SMA expression, a marker of myofibroblast differentiation, was significantly greater in wounds treated with Adipo^si−*Cont*^-EVs (Figures 5J,K), supporting Carm1’s role in promoting myofibroblast activation and wound contraction.

To confirm that the observed effects were not influenced by changes in glucose metabolism, blood glucose levels were monitored at days 0, 4, and 9 post-injury. As shown in Supplementary Figure S3, no significant differences were observed in blood glucose levels between the Adipo^si−*Cont*^-EVs and Adipo^si−*Carm1*^-EVs groups at any time point, suggesting that the treatments did not affect blood glucose levels during the course of the experiment.

### Exosomal Carm1 inhibits inflammation and promotes angiogenesis in the wound sites of diabetic mice

**FIGURE 6:**
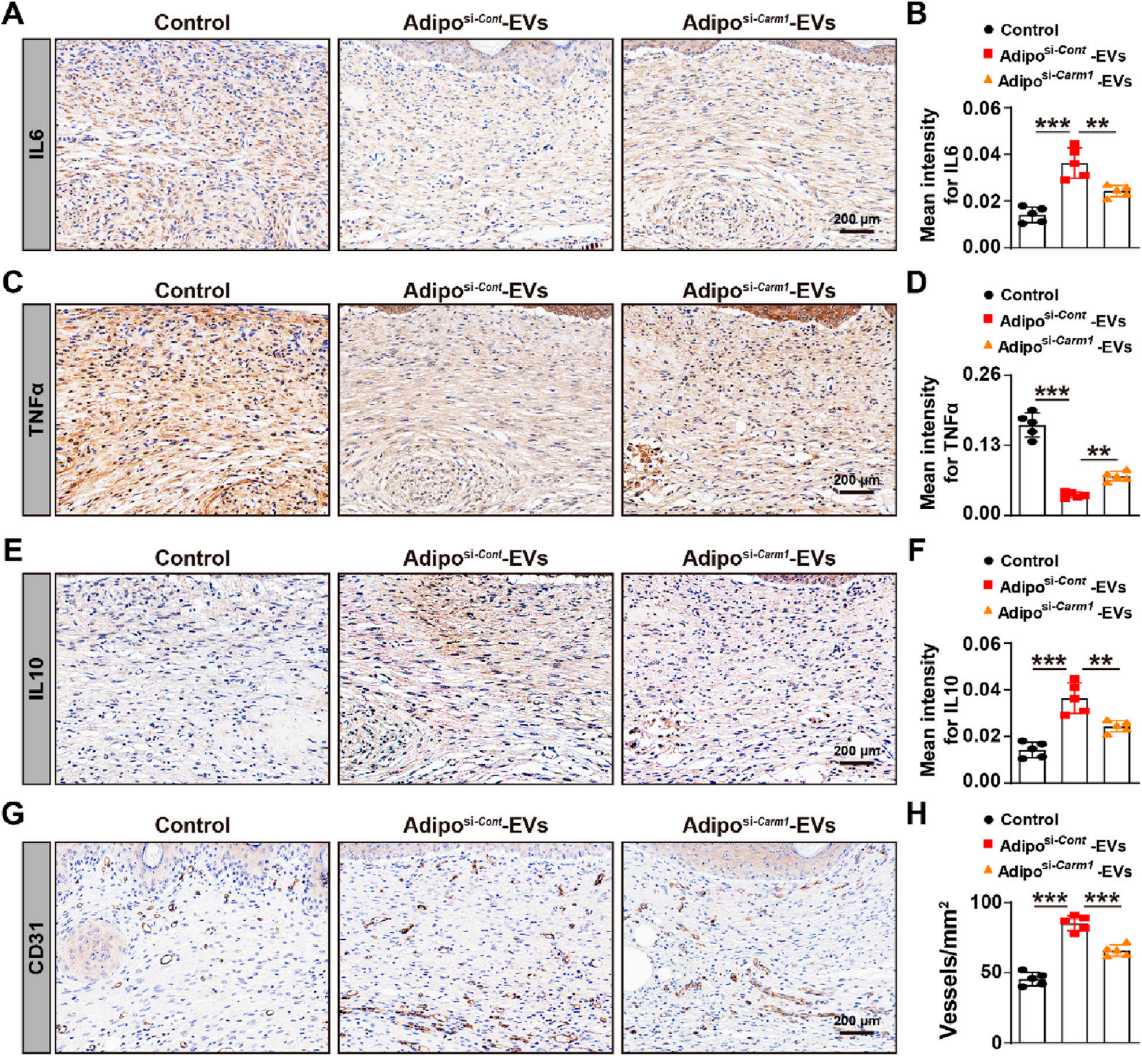
Exosomal Carm1 inhibits inflammation and promotes angiogenesis in mouse wound sites **(A,B)** IHC staining for IL6 in wound tissues from mice treated with Adipo-EVs (si-*Cont* or si-*Carm1*). Quantification of IL6 **(B)** shows significantly higher IL6 intensity in the si-*Carm1* group. N = 5 per group. **(C,D)** IHC staining for TNFα in wound tissues, with quantification **(D)** revealing increased TNFα intensity in the si-*Carm1* group, suggesting heightened inflammation. N = 5 per group. **(E,F)** IHC staining for IL10 in wound tissues. Quantification **(F)** demonstrates lower IL10 levels in the si-*Carm1* group, indicating a reduced anti-inflammatory response when Carm1 is knocked down. N = 5 per group. **(G,H)** IHC staining for CD31, an endothelial marker, illustrating fewer blood vessels in wounds treated with si-*Carm1* Adipo-EVs. Quantification **(H)** confirms significantly decreased microvessel density in the si-Carm1 group. N = 5 per group. Data are plotted as mean ± SD. ^*^ *P* < 0.05, ^**^ *P* < 0.01, ^***^ *P* < 0.001.

To investigate the effect of exosomal Carm1 on inflammation and angiogenesis, we assessed the expression of inflammatory cytokines and angiogenic markers in the wound sites of mice treated with Adipo^si−*Cont*^-EVs or Adipo^si−*Carm1*^-EVs. IHC evaluated inflammatory cytokine expression for IL6 (Figures 6A,B), TNFα (Figures 6C,D), and IL10 (Figures 6E,F). The mean intensity of IL6 (Figure 6B) and TNFα (Figure 6D) was significantly higher in the wounds treated with Adipo^si−*Carm1*^-EVs compared to those treated with Adipo^si−*Cont*^-EVs. In contrast, IL10 expression (Figure 6F), which is an anti-inflammatory cytokine, was significantly reduced in the Adipo^si−*Carm1*^-EVs group compared to the Adipo^si−*Cont*^-EVs group, suggesting that Carm1 plays a role in regulating inflammation at the wound site by inhibiting pro-inflammatory responses. IHC assessed angiogenesis for CD31 (Figures 6G,H), a marker of endothelial cells and blood vessels. Quantification of CD31-positive vessels (Figure 6H) showed significantly fewer blood vessels in the wounds treated with Adipo^si−*Carm1*^-EVs compared to those treated with Adipo^si−*Cont*^-EVs, indicating that the presence of Carm1 in Adipo-EVs promotes angiogenesis in the wound healing process. Together, these findings suggest that exosomal Carm1 inhibits inflammation by modulating the expression of pro-inflammatory cytokines (IL6 and TNFα), promoting anti-inflammatory cytokine (IL10) expression, and facilitating angiogenesis, contributing to a more efficient wound healing process.

## Discussion

This study demonstrates that adipocyte-derived exosomes (Adipo-EVs) significantly enhance wound healing in a diabetic model. Treatment with Adipo-EVs accelerated wound closure and improved tissue regeneration, as shown by narrower scars, increased collagen deposition, and a higher density of proliferating cells in the wound bed. A key finding is that Adipo-EVs exert dual pro-healing effects: anti-inflammatory and pro-angiogenic. Wounds treated with Adipo-EVs showed significant reductions in pro-inflammatory cytokines (IL-6 and TNF-α) and an increase in the anti-inflammatory cytokine IL-10. This indicates that Adipo-EVs help resolve excessive inflammation, creating a more favorable healing environment. Additionally, Adipo-EVs promoted neovascularization, reflected by enhanced endothelial tube formation *in vitro* and a higher number of CD31^+^ blood vessels *in vivo*. The combined suppression of inflammation and stimulation of angiogenesis likely contributed to the observed improvements in wound healing. Together, these results suggest that Adipo-EVs “re-balance” the wound microenvironment–dampening chronic inflammation and enhancing vascular supply for tissue repair.

Importantly, we identified Carm1 as a central mediator of these effects. Proteomic analysis revealed Carm1 to be highly enriched in Adipo-EVs, while another abundant protein, IGHE1, was not functionally relevant, as its signal disappeared after additional density-gradient purification, suggesting serum carry-over. Carm1 regulates critical pathways for inflammation resolution and vascular growth, making it the focus for further validation. Functional experiments showed that knocking down Carm1 in adipocytes (producing Carm1-depleted exosomes) markedly diminished their therapeutic efficacy. *In vitro*, Carm1-deficient Adipo-EVs lost much of their anti-inflammatory activity (failing to suppress IL-6/TNF-α or induce IL-10 in macrophages) and pro-angiogenic activity (endothelial cells formed fewer capillary-like structures). *In vivo*, Carm1-depleted Adipo-EVs resulted in slower wound closure and inferior healing compared to control Adipo-EVs. These findings strongly support that Carm1 is a key effector in Adipo-EVs and plays an essential role in suppressing inflammation and promoting angiogenesis during wound repair. This is the first demonstration that exosomal Carm1 can facilitate tissue healing, revealing a novel connection between an adipocyte-derived epigenetic enzyme and wound repair.

Our results align with a growing evidence that exosomes from various cell sources promote wound healing. Cell-derived exosomes as pro-regenerative agents are emerging in regenerative medicine, and our work with adipocyte exosomes extends this concept. Previous studies have shown that exosomes from mesenchymal stem cells (MSCs) can enhance cutaneous wound healing, particularly in diabetic wounds, by modulating inflammation, promoting angiogenesis, and stimulating dermal cells proliferation of (Xiao et al., 2019). Exosomes from adipose-derived MSCs (ADSCs) have been reported to accelerate diabetic wound healing by influencing extracellular matrix remodeling and reducing scar formation (Wang et al., 2017). Similarly, exosomes from M2-polarized macrophages (anti-inflammatory phenotype) promote angiogenesis and tissue regeneration in diabetic wounds by transferring miR-21 to endothelial cells and activating pro-angiogenic AKT/mTOR signaling (Lyu et al., 2022). Dermal fibroblast-derived exosomes also improve re-epithelialization, collagen deposition, and angiogenesis while inhibiting local inflammation (Han et al., 2021). Likewise, exosomes from human limbal epithelial cells were shown to modulate proliferation and migration of corneal stromal cells under both diabetic and non-diabetic conditions, further underscoring the broad regenerative potential of EV cargo (Verma et al., 2023). This led to faster and more effective wound closure (Han et al., 2021). Compared to these well-studied sources, our work is the first to showcase exosomes from mature adipocytes as a potent wound-healing agent. Adipocytes have not been widely considered in the wound-healing exosome paradigm; our findings highlight that adipocyte exosomes can be just as effective as those from stem cells or immune cells in driving key regenerative processes. This is an important addition to the field, given that adipocytes are abundant and easy to obtain. Our Adipo-EV therapy achieved outcomes (enhanced angiogenesis, reduced inflammation, improved healing) that are highly comparable to the effects reported for MSC- or macrophage-exosome therapies (Lyu et al., 2022; Han et al., 2021). Thus, we provide proof-of-concept that even terminally differentiated cells like adipocytes can produce exosomes with significant pro-healing bioactivity.

A novel aspect of our study is the identification of Carm1 as an exosomal factor mediating wound repair. Carm1 (PRMT4), a histone arginine methyltransferase, regulates gene expression as a nuclear co-activator. It plays roles in metabolic regulation and inflammation by coactivating PPARγ to promote adipocyte differentiation (Yadav et al., 2008) and modulating macrophage activation and T-cell differentiation. However, Carm1 has not been linked to extracellular vesicles or tissue regeneration. Our finding that adipocyte exosomes deliver Carm1 to target cells opens new perspective on exosomal cargo function. We hypothesize that Carm1, once transferred to recipient wound cells (macrophages or endothelial cells), may reprogram transcriptional programs to favor a pro-healing phenotype, downregulating NF-κB–dependent inflammatory genes and upregulating pro-angiogenic factors. This is consistent with our observation that without exosomal Carm1, macrophages showed higher IL-6/TNF and lower IL-10, and endothelial cells formed fewer vessels. Carm1’s role as an epigenetic enzyme suggests that Adipo-EVs may reprogram cells at the chromatin level, achieving sustained gene expression expression effects that benefit healing. The discovery of Carm1 as a critical exosomal component offers a specific molecular target to enhance or mimic Adipo-EV effects and demonstrates that exosomal proteins (not just microRNAs) can substantially influence wound healing.

Beyond mechanistic insights, our work has broader biological and translational implications. Adipocyte-derived exosomes could represent a new, readily accessible class of cell-free therapeutic agents for chronic wounds. Adipose tissue, often considered surgical waste, could be a valuable resource for regenerative medicine: a small volume of fat could be harvested and processed to isolate Adipo-EVs for autologous treatment, bypassing immune rejection and ethical concerns related to stem cell therapies (Maranda et al., 2017; Cao et al., 2017). Compared to live cells therapies, exosomes are safer, cannot form tumors or unwanted cell lineages, and are easier to store and standardize. Another advantage is their unique cargo. Adipocytes secrete factors like adiponectin, which is anti-inflammatory and pro-angiogenic and has been linked to better wound healing outcomes in diabetics. We showed that adiponectin is present in Adipo-EVs ​and may contribute to cytokine changes and vessel growth. The protein and RNA content of Adipo-EVs is enriched in regulators of metabolism, angiogenesis, and immune responses, making them particularly suited to address the multifactorial nature of chronic wounds by targeting different aspects of the wound microenvironment. From a clinical standpoint, adipocyte exosomes could be applied topically or via local injection to chronic wounds. They could also potentially be incorporated into biomaterials–for example, loaded into a hydrogel or scaffold that is placed in the wound bed. Such an approach has been tested with MSC-derived exosomes on an acellular dermal scaffold, yielding improved wound healing by sustained release of exosomes at the injury site (Xiao et al., 2021). Adipo-EVs may be integrated into wound dressings to enhance their retention and efficacy. Overall, our findings add to the growing evidence that exosome-based therapies can achieve meaningful tissue repair, and they introduce adipocyte exosomes as a promising new tool in this arena.

Despite the encouraging results, several limitations of our study should be acknowledged. First, our *in vivo* experiments were conducted in a murine model of diabetic wound healing. Murine skin and wound-healing physiology differ from humans in aspects like skin structure and immune response, so the efficacy of Adipo-EVs will need validation in human tissues or more complex models. Additionally, the diabetic mice used in this study (if induced by streptozotocin or genetic models) may not capture all features of human diabetic ulcers, such as neuropathy or repetitive trauma. Second, we harvested exosomes cultured from adipocytes under normal (non-diabetic) conditions. It is known that the metabolic state of adipocytes can influence their secretory profile–for example, adipocytes from obese or diabetic donors release factors that can be detrimental to healing (Zhang et al., 2019). It will be essential to determine whether Adipo-EVs derived from diabetic adipose tissue have the same beneficial effects or if certain pathological cargo (like the circRNA reported by Zhang et al.) (Zhang et al., 2019) might counteract their therapeutic potential. Another limitation is the isolation and dosing of exosomes. We used ultracentrifugation to isolate Adipo-EVs and characterized them by standard markers, but exosome preparations can be heterogeneous and contain various vesicle subtypes or protein contaminants. Achieving clinical-grade purity and consistent potency will require process optimization. Likewise, the optimal dosage and frequency of Adipo-EV administration remain to be determined–too low a dose might be ineffective, while too high could have unforeseen off-target effects. We also cannot rule out that some effects of Adipo-EVs are indirect; for instance, they might act on 1 cell type that influences others (e.g., modulating macrophages that then promote angiogenesis via their secreted factors). While insightful, our mechanistic focus on Carm1 does not exclude the contribution of other cargo. Adipo-EVs contain hundreds of proteins and RNAs, and multiple factors likely act in concert to produce the full pro-healing effect. We observed that another protein (IGHE) was highly enriched in Adipo-EVs, and there are undoubtedly various microRNAs packaged in these vesicles that have known roles in wound healing. Our study primarily centered on Carm1, so the roles of these other components remain unexplored. This presents an opportunity for future research but is currently a limitation in fully understanding how Adipo-EVs function.

We see several future directions and applications prompted by our findings. An immediate next step is to investigate the therapeutic use of Adipo-EVs in more clinically relevant models. This could include testing on human skin explants or in a larger animal model (such as diabetic pigs, whose skin more closely resembles human skin). Such studies would provide valuable data on dosing, administration methods, and safety in a setting closer to human wounds. Scaling up Adipo-EV production is another practical consideration–methods like tangential flow filtration or size-exclusion chromatography could be employed to isolate exosomes more efficiently than ultracentrifugation, facilitating larger-scale preparations for trials. It would also be interesting to compare Adipo-EVs from healthy versus diabetic or obese adipose tissue to characterize differences in cargo. If diabetic Adipo-EVs are less effective, one could envision “engineering” exosomes for therapy, for example, by overexpressing beneficial molecules (such as Carm1 or specific miRNAs) in donor adipocytes before exosome isolation to enhance their reparative potency. Bioengineering approaches could further improve delivery, as mentioned–integrating Adipo-EVs into biocompatible scaffolds or hydrogels to localize and prolong their action in wounds (Xiao et al., 2021). Another avenue is exploring the application of Adipo-EVs to other types of tissue repair. Chronic skin wounds are an obvious target, but adipocyte exosomes might also aid in healing different tissues that involve angiogenesis and inflammation, such as ischemic cardiac or limb tissue or even bone healing in diabetics. Finally, mechanistic studies should investigate how Adipo-EVs reprogram the wound environment. Our identification of Carm1 is a start; future work could perform RNA sequencing or epigenetic analyses of wound macrophages/endothelial cells treated with Adipo-EVs to pinpoint the gene networks altered by exosomal delivery. Unraveling these pathways could uncover new drug targets or biomarkers of healing response. In conclusion, this study provides new evidence that adipocyte-derived exosomes are potent modulators of wound repair, suppressing chronic inflammation and inducing angiogenesis in diabetic wounds. By leveraging a naturally abundant cell type and its secreted vesicles, we introduce a potentially translatable cell-free therapy and identify Carm1 as a novel molecular player in exosome-facilitated healing. With further development and validation, Adipo-EV-based treatments could become a valuable addition to the arsenal for managing chronic diabetic wounds and improving patient outcomes.

## Data Availability

The data presented in this study are deposited in the Figshare repository, accession number https://doi.org/10.6084/m9.figshare.29878838.v1.
