# Supplementary material for: Exosomal coactivator-associated arginine methyltransferase 1 derived from adipocytes accelerates diabetic wound healing by modulating inflammation and promoting angiogenesis
Source: Front Bioeng Biotechnol. 2025 Aug 21;13:1610806. doi: 10.3389/fbioe.2025.1610806 (PMC12408491; doi:10.3389/fbioe.2025.1610806)
Supplement: Supplementary file 1 [file Image1.PDF]

Supplementary Materials

The procedure for forming a skin wound

Full-thickness excisional skin wounds were created on the backs of 6- to 8-week-old diabetic mice. Mice were first anesthetized with an intraperitoneal injection of 50 mg/kg pentobarbital sodium, and their dorsal fur was shaved. Using a sterile surgical punch (12 mm in diameter), two full-thickness wounds were created on the dorsum of each mouse. The wound edges were carefully separated, and the wounds were left open to heal naturally. Wound healing was then monitored and assessed at multiple time points (days 1, 3, 5, 7, and 9 post-wounding). The procedure was performed under sterile conditions, and the animals were closely monitored throughout the experiment.

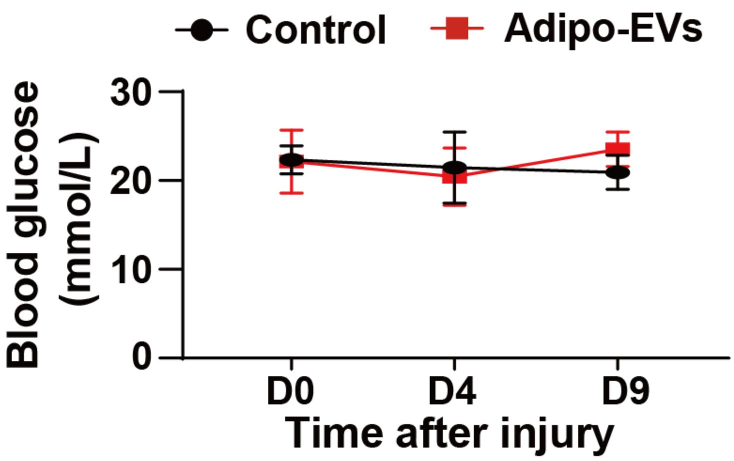

Figure S1. Blood glucose levels in diabetic mice treated with Adipo-EVs. Blood glucose measurements were taken at days 0, 4, and 9 post-injury.

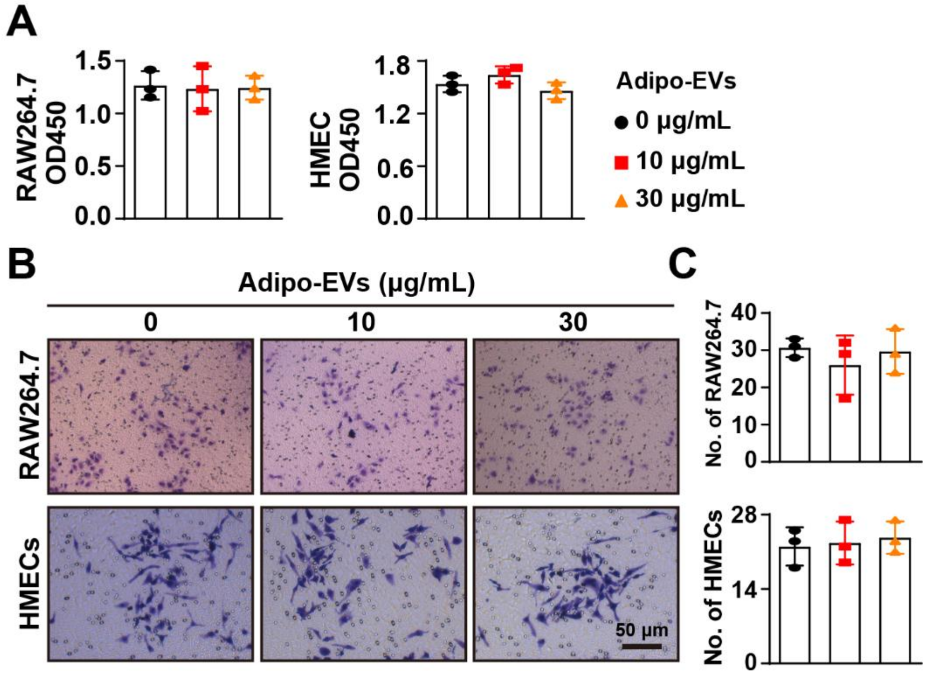

Figure S2. Effect of Adipo-EVs on cell proliferation and migration. (A) CCK-8 assay showing the proliferation of RAW264.7 macrophages and HMECs treated with different concentrations of Adipo-EVs (0, 10, and 30 μg/mL). No significant difference in cell proliferation was observed at the tested concentrations. **(B)** Transwell migration assay images showing the migration of RAW264.7 and HMECs after treatment with

Adipo-EVs (0, 10, and 30 μg/mL). Representative images are shown. **(C)** Quantification of the number of migrated RAW264.7 and HMECs. No significant effects on cell migration were observed at the tested concentrations of Adipo-EVs. Data are presented as mean ± SD (n = 3). Scale bar = 50 μm.

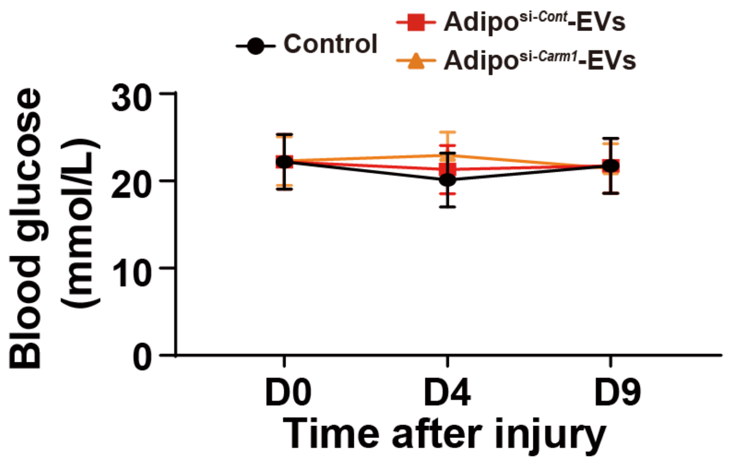

Figure S3. Blood glucose levels in diabetic mice treated with Adipo-EVs or Adipo-EVs from si-*Carm1* adipocytes. Blood glucose measurements were taken at days 0, 4, and 9 post-injury.
